# The Impact of Vascular Management on Postoperative Complications in Patients Undergoing Surgery for Retroperitoneal Leiomyosarcoma

**DOI:** 10.3390/curroncol33020090

**Published:** 2026-02-02

**Authors:** Neha Malik, Seokhun Kim, Christopher P. Scally, Emily Z. Keung, Heather Lillemoe, Keila E. Torres, Kelly K. Hunt, Sophia Khan, Christina L. Roland, Heather G. Lyu

**Affiliations:** 1 Department of Surgical Oncology, The University of Texas MD Anderson Cancer Center, Houston, TX 77030, USA; neha.malik@uth.tmc.edu (N.M.); cpscally@mdanderson.org (C.P.S.); ekeung@mdanderson.org (E.Z.K.); halillemoe@mdanderson.org (H.L.); ketorres@mdanderson.org (K.E.T.); khunt@mdanderson.org (K.K.H.); clroland@mdanderson.org (C.L.R.); 2 Center for Clinical Research and Evidence-Based Medicine, McGovern Medical School, Houston, TX 77054, USA; seokhun.kim@uth.tmc.edu; 3 Department of Vascular Surgery, The University of Texas MD Anderson Cancer Center, Houston, TX 77030, USA; khans27@upmc.edu

**Keywords:** vascular, retroperitoneal sarcoma, surgery, complications

## Abstract

Background: Retroperitoneal leiomyosarcomas are aggressive malignancies. Complete surgical resection with negative margins is crucial to decrease the risk of recurrence but can be risky due to vascular involvement. The aim of our study was to evaluate the different approaches to IVC and renal vein management and their impact on postoperative complications. Methods: We performed a retrospective review of patients who underwent surgery for retroperitoneal leiomyosarcoma with IVC and/or renal vein involvement at our institution from 2016 to 2024. Patients were stratified by intraoperative vascular management, including ligation only versus varying forms of vascular reconstruction. Postoperative complications, including bleeding, transfusions, the need for acute and chronic hemodialysis, and thromboembolic events, were recorded. Chi-squared tests were used to compare rates of postoperative complications by vascular management. A *p*-value of 0.05 was considered statistically significant. Results: We identified 60 patients at our institution who underwent surgery for leiomyosarcoma with IVC and/or renal vein involvement. Ten patients underwent IVC ligation alone due to thrombosis, thirty-six had IVC replacement, and fourteen had patch angioplasty. In the entire cohort, twenty-six patients (43.3%) experienced an adverse event after surgery. When looking at postoperative adverse events by IVC management, we did not find any statistically significant differences among rates of adverse events by group. There were also no statistically significant differences in complications following renal vein ligation versus renal vein reconstruction. Conclusions: Patients with leiomyosarcoma with IVC and/or renal vein involvement have several options for intraoperative vascular management. Our data demonstrates that there are no statistically significant differences in rates of complications among the different groups.

## Simple Summary

Retroperitoneal sarcomas are an aggressive form of cancer that may involve major blood vessels in the abdomen. Our study looked at how surgeons managed involved blood vessels and whether that impacted complications after surgery. We found that there were no significant differences in the rates of complications after surgery when looking at how the involved blood vessels were managed.

## 1. Introduction

Vascular leiomyosarcomas are a distinctive subgroup of sarcomas that originate in the smooth muscle of blood vessels. Retroperitoneal leiomyosarcomas often involve the inferior vena cava (IVC), renal veins, and gonadal veins and are the second most common type of retroperitoneal sarcomas [1]. Complete surgical resection remains the cornerstone of treatment, and obtaining negative margins is crucial to decrease the risk of recurrence [2,3]. Curative intent resection can be challenging when critical vascular structures are involved or encased by the tumor [1]. Potential postoperative complications include but are not limited to significant hemorrhage, thromboembolic events, infection, and organ dysfunction, including renal failure.

Vascular reconstruction after resection of IVC leiomyosarcoma often involves vein reconstruction versus ligation. The anatomic location of the tumor in relation to the IVC and its major tributaries can impact the choice of management. Patients with zone 2 IVC leiomyosarcomas that involve the renal veins may require more complex planning [4]. These patients may be at higher risk for the development of postoperative acute kidney injury (AKI) and chronic kidney injury (CKD) that can potentially impact their ability to receive adjuvant therapy [5]. Both surgical options carry long-term effects after surgery, and patients should be counseled on the potential impacts of vascular reconstruction, such as long-term anticoagulation, versus the long-term complications associated with vascular ligation, including vascular congestion.

Currently, no guidelines exist for the surgical management of IVC leiomyosarcomas with vascular involvement. Due to the rarity of IVC leiomyosarcomas, there is a paucity of data on the impact of vascular management on postoperative outcomes. Factors that may influence a surgeon’s decision for vascular reconstruction versus ligation include tumor location and renal vein involvement, the extent of IVC resection required, and the presence of collateral venous drainage at the time of surgery [5,6,7]. The aim of our study was to evaluate the different approaches to IVC and renal vein management and their impact on postoperative complications. The findings of this study may inform surgical decision-making and optimize perioperative and postoperative management to improve outcomes for patients with IVC leiomyosarcomas.

## 2. Methods

### 2.1. Study Overview

This study was approved by the Institutional Review Board at MD Anderson Cancer Center. A retrospective review of patients who underwent surgery for retroperitoneal leiomyosarcoma with IVC involvement and with or without renal vein involvement at our institution from April 2016 to March 2024 was performed.

### 2.2. Data Collection

Patient demographic and clinical data were collected. Surgical variables included intraoperative management of the primary tumor, vascular surgery management, and renal vein involvement. Postoperative complications were recorded for each patient, including the need for postoperative transfusion, the need for acute or chronic dialysis, the diagnosis of acute kidney injury (AKI) defined using the Kidney Disease: Improving Global Outcomes criteria, the diagnosis of postoperative chronic kidney disease (CKD) defined as GFR < 60 within 90 days, postoperative bleeding events, clinically significant thromboembolic events, or the need for any type of reintervention. Anticoagulation use at 1–3 months post-surgery and 1 year post-surgery was collected for all patients. Patients were stratified by intraoperative IVC management, including ligation only versus varying methods of vascular reconstruction, and postoperative complications were compared across the groups. Patients with leiomyosarcomas with renal vein involvement were stratified by renal vein ligation, primary renal vein reconstruction, or graft reconstruction, and postoperative outcomes were compared.

### 2.3. Outcomes

The primary outcome assessed was any adverse event occurring after surgery. A categorical composite outcome variable was created to represent the occurrence of any major adverse event after surgery. Adverse events included postoperative bleeding, thromboembolic events, or the need for reintervention. Reintervention included any surgical or interventional radiology procedure. Secondary outcomes included the need for acute or chronic hemodialysis (HD) and the diagnosis of postoperative acute kidney injury (AKI).

### 2.4. Statistical Analysis

Descriptive statistics were used to describe the overall cohort and subgroups. Chi-squared tests were used to compare rates of categorical postoperative complications across groups. Kruskal–Wallis and ANOVA tests were used to compare continuous variable postoperative outcomes. A *p*-value of <0.05 was considered statistically significant. To balance patients between the three vascular management groups, a propensity score analysis was performed on all patients. Patients were grouped according to their vascular management strategy, and a propensity score model was fitted to derive weights. Variables included in the propensity score model included age, history of smoking, hypertension, kidney disease, coronary artery disease, hyperlipidemia, preoperative DVT or PE, and preoperative IVC occlusion. Balances were checked before and after applying weights to ensure that the balance improved after the weights were applied. This weighted sample was used to perform a multivariate logistic regression model to look at the impact of vascular management on the odds of having any adverse outcome after surgery, along with the odds of developing an AKI or needing postoperative hemodialysis (HD).

## 3. Results

### 3.1. Demographics and Clinical Presentation

Sixty patients were identified during the study period who were diagnosed with IVC leiomyosarcoma and underwent surgical resection. The mean age was 57.1 (SD: 12.1), and 61.7% (*n* = 37) of our cohort was female. Table 1 lists all demographics and clinical characteristics of the study cohort. Of note, 53.3% (*n* = 32) of patients had pre-existing hypertension, and 6.7% (*n* = 4) had pre-existing kidney disease. The majority of patients (73.3%) underwent preoperative chemotherapy and less than half (41.7%) underwent pre-operative radiation. IVC occlusion was rare in the overall cohort but more commonly seen in the IVC ligation group (*n* = 10; 16.7%).

### 3.2. Operative Management

Ten patients were treated with IVC ligation (16.7%), thirty-six underwent IVC replacement using polytetrafluoroethylene (PTFE) or Dacron grafts (60%), and fourteen underwent patch angioplasty using bovine pericardium (23.3%). Thirteen patients (21.7%) had a nephrectomy, with no significant difference between IVC management groups (*p* = 0.773). Twenty-two patients had renal vein involvement. Of the twenty-two patients, thirteen (59.1%) had renal vein ligation alone, three (13.6%) had primary reconstruction of the renal vein, and six (27.3%) underwent graft reconstruction.

### 3.3. Postoperative Outcomes

Twenty (33.3%) patients experienced the primary outcome of a major adverse event after surgery. Four patients (6.7%) experienced a bleeding event, eleven (18.3%) experienced a thromboembolic event, and seven (11.7%) required some form of reintervention. There were no patient deaths within 30 days from surgery. Although there were no statistically significant differences in adverse events among the IVC management groups (*p* = 0.225), four (40%) patients who underwent the IVC ligation experienced a major adverse event. All four patients in the IVC ligation group who experienced a major adverse event were found to have a thromboembolic event during their postoperative period, with one patient also requiring interventional radiology reintervention for a drain placement. Patients who underwent IVC replacement or patch angioplasty had adverse events more evenly distributed among bleeding, thromboembolic events, and reintervention. Table 2 lists all adverse events stratified by IVC management strategy.

The 22 patients who had renal vein involvement were also stratified into groups based on renal vein management. When comparing the incidence of postoperative complications across groups, there were no statistically significant differences based on renal vein management, but three (50%) patients who underwent graft reconstruction experienced a major adverse event (Table 3). Importantly, no patients who underwent renal vein ligation or reconstruction required chronic HD after surgery, and only two patients with renal vein involvement required acute HD. Both of these patients had no prior history of kidney disease, but they both underwent nephrectomies as part of their resection.

A multivariate logistic regression model using patients from the propensity score analysis was performed to assess the odds of developing a major adverse event after surgery by IVC management strategy (Table 4). When looking at the primary outcome of any major adverse event after surgery, IVC replacement and patch angioplasty were associated with lower odds compared to IVC ligation (*p* < 0.001). Both IVC replacement and patch angioplasty were also associated with lower odds of developing postoperative AKI and needing postoperative hemodialysis compared to IVC ligation alone.

### 3.4. Postoperative Anticoagulation

At one to three months after their initial surgery, the majority of patients (46.7%) were either prescribed aspirin or no form of anticoagulation. This increased to 58.3% of patients at their one-year postoperative point. There were no statistically significant differences among anticoagulation use after surgery at either the one-to-three-month or one-year time point for any of the IVC management groups. Anticoagulation use also significantly varied among patients, with no standardized approach based on IVC management or patient comorbidities (Supplemental Table S1).

## 4. Discussion

This study provides the largest comprehensive overview of the various intraoperative vascular techniques of patients with IVC leiomyosarcomas and the associated postoperative complications. Given the rarity of this disease and the technical complexity of surgical resection, evidence guiding optimal perioperative vascular management remains limited. Several key findings emerged from our analysis, offering important insights into the role of vascular ligation versus reconstruction during complex sarcoma resections and underscoring the need for individualized, anatomy-driven decision-making in this challenging population.

We found that 33% of all patients in our cohort experienced a major adverse event after surgery, which included bleeding, thromboembolic events, or the need for reintervention. This complication rate is consistent with previously reported morbidity rates of 25–40% in contemporary institutional series [5,8]. While there were no statistically significant differences when comparing the incidence between IVC management groups, distinct patterns emerged. Patients who underwent IVC ligation only experienced thromboembolic events, including DVT and renal vein thrombus, as their major postoperative adverse event, with one patient requiring reintervention. IVC replacement and patch angioplasty patients experienced major adverse events more evenly distributed among the three types of adverse events, with patch angioplasty patients experiencing the fewest major adverse events after surgery.

The higher thromboembolic risk observed in the IVC ligation cohort likely reflects baseline vascular pathology rather than operative technique alone. Patients selected for IVC ligation were more often found to have pre-existing IVC occlusion, consistent with prior observations that chronic venous obstruction leads to the development of extensive collateral circulation [9,10]. Chronic IVC occlusion permits gradual physiologic adaptation and collateral venous drainage, which may mitigate acute hemodynamic consequences following ligation but predispose patients to postoperative venous thromboembolism due to stasis-induced vein wall injury and possible hypoxemia-induced tissue factor upregulation [11].

Despite the higher incidence of thromboembolic events among patients undergoing IVC ligation, we did not observe increased anticoagulation use in this patient population at the one-to-three-month point or one-year point after surgery. This finding highlights an important gap in standardized postoperative management for patients undergoing major venous ligation. Current cancer-associated thrombosis guidelines provide general recommendations for anticoagulation but do not specifically address patients with intentional IVC ligation, leaving postoperative surveillance and anticoagulation strategies largely institution dependent. While these findings still may not confirm that the choice of vascular techniques for IVC management independently drives the risk of postoperative complications in this population, they can better inform how we manage these patients in the postoperative period. Future studies focused on the thromboprophylaxis optimization in this high-risk population are warranted.

For patients whose tumors involved the renal vein, specifically the left renal vein, there were similarly no differences in postoperative complications among those who underwent renal vein ligation, primary reconstruction, or graft reconstruction. Importantly, there was no significant difference in the rates of chronic kidney disease between these groups. This finding is particularly relevant in the setting of surgical concerns regarding renal function preservation in cases where the renal vein is involved. Our data suggest that, in select patients with the appropriate anatomy, renal vein ligation is a safe option without a disproportionate risk of developing CKD, challenging the assumption that complex reconstructions are always necessary to preserve kidney function. This finding reflects one’s capacity to compensate through collateral circulation and supports the more minimal strategy where renal vein involvement is present. Our findings align with prior studies demonstrating preserved renal function following left renal vein ligation due to robust collateral drainage through the gonadal, adrenal, and lumbar venous systems [12,13].

Our study had several limitations. The retrospective nature of this study exposes it to the risk of selection bias and confounding. Specifically, we found that patients who had pre-existing IVC occlusion were more likely to undergo IVC ligation, which may have influenced both surgical decision-making and postoperative outcomes. Additionally, given that IVC leiomyosarcoma is a rare disease, we had a very small sample size of only 60 patients. Despite our small sample size, our study cohort at our institution represents one of the larger cohorts of IVC leiomyosarcoma patients. Future multi-institutional studies are underway to further elucidate the long-term functional outcomes associated with these vascular strategies and to refine the indications for ligation versus reconstruction.

Overall, our findings provide evidence to support the various management strategies of IVC and renal vein involvement during resection of retroperitoneal leiomyosarcomas. Despite the complexity of these cases, more aggressive vascular reconstruction may not always confer a benefit in reducing complications, and ligation can be considered a safe and effective approach in select patients [2]. While our data suggest that more aggressive vascular reconstruction may not consistently reduce postoperative complications and that ligation can be considered a safe option in select patients, these conclusions are tentative rather than definitive. An individualized, patient-centered approach to vascular techniques is paramount in the surgical management of IVC leiomyosarcomas. Future studies should focus on multi-institutional collaboration to validate these findings and to develop consensus guidelines for complex vascular management.

## 5. Conclusions

Our data demonstrates that in the IVC leiomyosarcoma patient cohort, vascular management does not significantly impact the rate of postoperative complications. Patients with renal vein involvement may undergo renal vein ligation without significant impact on long-term kidney function. Larger, prospective trials are needed to further understand the impact of vascular management on postoperative outcomes in IVC leiomyosarcoma patients.

## Figures and Tables

**Table 1:** Cohort demographics and clinical characteristics.

**Demographics** **(*n* = 60)**	
Age (mean, SD)	57.1 (12.1)
**Gender**	
Male	23 (38.3%)
Female	37 (61.7%)
**Race/Ethnicity**	
Caucasian	38 (63.3%)
Hispanic	14 (23.3%)
African American	5 (8.3%)
Asian	2 (3.3%)
Arab	1 (1.7%)
Smoking History	18 (30%)
**Chronic Conditions**	
Diabetes	7 (11.7%)
Hypertension	32 (53.3%)
Coronary Artery Disease	6 (10%)
Hyperlipidemia	11 (18.3%)
Kidney Disease	4 (6.7%)
Preoperative Chemotherapy	44 (73.3%)
Preoperative Radiation	25 (41.7%)
Preoperative DVT/PE	15 (25%)
Preoperative Antithrombotic	26 (43.3%)
Estimated Blood Loss During Surgery (mean (SD), days)	2091 (3576.2)
Length of Hospital Stay (mean, SD)	7.6 (5.4)
Nephrectomy Performed	13 (21.7%)
Renal Vein Involvement	22 (36.7%)
**Renal Vein Management**	
Renal Vein Ligation	13 (21.7%)
Renal Vein Primary Reconstruction	3 (5%)
Renal Vein Reconstruction with Graft	6 (10%)
**IVC Management**	
IVC Ligation	10 (16.7%)
IVC Replacement	36 (60%)
Patch Angioplasty	14 (23.3%)

**Table 2:** Comparison of postoperative complications by IVC management.

	IVC Ligation (*n* = 10)	IVC Replacement (*n* = 36)	Patch Angioplasty (*n* = 14)	*p*-Value
AKI	4 (40%)	12 (33.3%)	3 (21.4%)	0.59
CKD	2 (20%)	5 (13.9%)	4 (28.6%)	0.48
Postop HD	1 (10%)	1 (2.8%)	2 (14.3%)	0.31
Chronic HD	0 (0%)	0 (0%)	0 (0%)	-
Major Postoperative AE	7 (70%)	15 (41.7%)	4 (28.6%)	0.12
Reintervention	1 (10%)	6 (16.7%)	0 (0%)	0.253
Postop Thromboembolic Event	4 (40%)	6 (16.7%)	1 (7.1%)	0.112
Postop Bleeding Event	0 (0%)	3 (8.3%)	1 (7.1%)	0.644
<30-day Mortality	0 (0%)	0 (0%)	0 (0%)	-
Length of Hospital Stay (Mean (SD), Days)	9.9 (7.9)	6.7 (2.4)	8.5 (8.0)	0.19

**Table 3:** Comparison of postoperative complications by renal vein management.

N (%)	Ligation Only (*n* = 13)	Primary Recon (*n* = 3)	Graft Recon (*n* = 6)	*p*-Value
AKI	4 (30.8%)	2 (66.7%)	3 (50%)	0.37
CKD	1 (7.7%)	1 (33.3%)	0 (0%)	0.32
Postop HD	2 (15.4%)	0 (0%)	0 (0%)	0.50
Chronic HD	0 (0%)	0 (0%)	0 (0%)	
Major Postoperative AE	7 (53.8%)	1 (33.3%)	4 (66.7%)	0.44
Reintervention	2 (15.4%)	0 (0%)	1 (16.7%)	0.86
Postop Thromboembolic Event	1 (7.7%)	1 (33.3%)	1 (16.7%)	0.65
Postop Bleeding Event	1 (7.7%)	0 (0%)	1 (16.7%)	0.72
<30-day Mortality	0 (0%)	0 (0%)	0 (0%)	-
Length of Hospital Stay (Median (SD), Days)	7 (7.6)	6 (6.4)	6 (4.4)	0.70

**Table 4:** Multivariate logistic regression.

	Odds Ratio	95% Confidence Interval	*p*-Value
**Odds of Major Adverse Event**			
IVC Ligation *	4.51	2.12–11.08	<0.001
IVC Replacement	0.12	0.04–0.31	<0.001
Patch Angioplasty	0.07	0.02–0.18	<0.001
**Odds of AKI**			
IVC Ligation *	3.88	1.87–9.07	<0.001
IVC Replacement	0.11	0.04–0.29	<0.001
Patch Angioplasty	0.06	0.02–0.17	<0.001
**Odds of Postoperative HD**			
IVC Ligation *	0.78	0.41–1.46	0.44
IVC Replacement	0.02	0.001–0.12	<0.001
Patch Angioplasty	0.22	0.07–0.59	<0.001

* Comparison group.

## Data Availability

The data supporting the reported results in this study are not publicly available due to privacy and ethical restrictions associated with the use of private patient data. However, we are open to sharing the data with other research institutions that may be interested in collaborating. Access to the data can be facilitated upon the completion of the necessary institutional agreements, including the appropriate Institutional Review Board (IRB) approvals, Material Transfer Agreements (MTAs), and Data Use Agreements (DUAs). Interested parties should contact us directly to initiate discussions regarding data access under these terms.

## Supplementary Materials

The following supporting information can be downloaded at: https://www.mdpi.com/article/10.3390/curroncol33020090/s1, Table S1: Anticoagulation Use After Surgery.
