# Supplementary material for: The Impact of Vascular Management on Postoperative Complications in Patients Undergoing Surgery for Retroperitoneal Leiomyosarcoma
Source: Curr Oncol. 2026 Feb 2;33(2):90. doi: 10.3390/curroncol33020090 (PMC12939976; doi:10.3390/curroncol33020090)
Supplement: Supplementary file 1 [file curroncol-33-00090-s001.zip › curroncol-3948049-supplementary.pdf]

Supplemental Table 1: Anticoagulation Use After Surgery

n (%)	Cohort (n=60)	IVC Ligation (n=10)	IVC Replacement (n=36)	Patch Angioplasty (n=14)	P value
**1-3 months Post-Op**					0.437
ASA/Nothing	28 (46.7)	3 (30)	15 (41.7)	10 (71.4)	
Prophylactic Lovenox	9 (15)	4 (40)	4 (11.1)	1 (7.1)	
Therapeutic Lovenox	5 (8.3)	1 (10)	3 (8.3)	1 (7.1)	
Eliquis	12 (20)	2 (20)	8 (22.2)	2 (14.3)	
Xarelto	3 (5)	0 (0)	3 (8.3)	0 (0)	
Coumadin	1 (1.7)	0 (0)	1 (2.8)	0 (0)	
Plavix	2 (3.3)	0 (0)	2 (5.6)	0 (0)	
**1 year Post-Op**					0.545
ASA/Nothing	35 (58.3)	7 (70)	20 (55.6)	8 (57.1)	
Prophylactic Lovenox	0 (0)	0 (0)	0 (0)	0 (0)	
Therapeutic Lovenox	0 (0)	0 (0)	0 (0)	0 (0)	
Eliquis	10 (16.7)	2 (20)	5 (13.9)	3 (21.4)	
Xarelto	2 (3.3)	1 (10)	1 (2.8)	0 (0)	
Coumadin	1 (1.7)	0 (0)	1 (2.8)	0 (0)	
Plavix	5 (8.3)	0 (0)	5 (13.9)	0 (0)	
Unknown	7 (11.7)	0 (0)	4 (11.1)	3 (21.4)	
